# Metabolites Profiling and Bioassays Reveal *Bassia indica* Ethanol Extract Protective Effect against Stomach Ulcers Development via HMGB1/TLR-4/NF-κB Pathway

**DOI:** 10.3390/antiox12061263

**Published:** 2023-06-12

**Authors:** Zeinab A. El-Gendy, Rehab F. Taher, Abdelbaset M. Elgamal, Ahmed Serag, Azza Hassan, Gehad A. Abdel Jaleel, Mohamed A. Farag, Abdelsamed I. Elshamy

**Affiliations:** 1 Department of Pharmacology, Medical Research and Clinical Studies Institute, National Research Centre, 33 El Bohouth St., Dokki, Giza 12622, Egypt; za.el-gendy@nrc.sci.eg (Z.A.E.-G.); ga.jaleel@nrc.sci.eg (G.A.A.J.); 2 Department of Natural Compounds Chemistry, National Research Center, 33 El Bohouth St., Dokki, Giza 12622, Egypt; rf.farid@nrc.sci.eg; 3 Department of Chemistry of Microbial and Natural Products, National Research Centre, 33 El-Bohouth St., Dokki, Giza 12622, Egypt; 4 Pharmaceutical Analytical Chemistry Department, Faculty of Pharmacy, Al-Azhar University, Cairo 11751, Egypt; ahmedserag777@azhar.edu.eg; 5 Department of Pathology, Faculty of Veterinary Medicine, Cairo University, Giza 12211, Egypt; azzahassa1999@cu.edu.eg; 6 Pharmacognosy Department, Faculty of Pharmacy, Cairo University, Kasr el Aini St., Cairo 11562, Egypt; mohamed.farag@pharma.cu.edu.eg

**Keywords:** gastroprotection, *Bassia indica*, HMGB1, NF-κB, TLR-4, IL-1β, Nrf2, UPLC chemical profile

## Abstract

Clinical manifestation of gastric ulcers is frequent, in addition to their costly drug regimens, warranting the development of novel drugs at lower costs. Although *Bassia indica* is well characterized for its anti-inflammatory and antioxidant potential, capacity of its ethanol extract (BIEE) to prevent stomach ulcers’ progression has not been reported. A nuclear protein termed high-mobility group box 1 (HMGB1) plays a key role in the formation of stomach ulcers by triggering a number of inflammatory responses. The main purpose of the current investigation was to evaluate the in vivo anti-inflammatory and anti-ulcerogenic capabilities of BIEE against ethanol-induced gastric ulcers in rats via the HMGB1/TLR-4/NF-B signaling pathway. HMGB1 and Nuclear factor kappa (NF-B) expression, IL-1β and Nrf2 contents showed an increase along with ulcer development, concurrent with an increase in immunohistochemical TLR-4 level. In contrast, pre-treatment with BIEE significantly reduced HMGB1 and Nuclear factor kappa (NF-B) expression levels, IL-1β and Nrf2 contents and ulcer index value. Such protective action was further confirmed based on histological and immunohistochemical TLR-4 assays. Untargeted analysis via UPLC-ESI–Qtof-MS has allowed for the comprehensive characterization of 40 metabolites in BIEE mostly belonged to two main chemical classes, *viz*., flavonoids and lipids. These key metabolites, particularly flavonoids, suggesting a mediation for the anti-inflammatory and anti-ulcerogenic properties of BIEE, pose it as a promising natural drug regimen for treatment of stomach ulcers.

## 1. Introduction

With a 10% frequency in human civilization, peptic ulcer disease (PUD) is a widespread gastrointestinal condition [1], which affects the stomach’s ability to balance aggressive and protective forces [2]. Along with exogenous factors such as alcohol consumption and the use of non-steroidal anti-inflammatory medicines, aggressive factors also include the production of pepsin and stomach acid, active free radicals and oxidants, leukotrienes, and endothelins [3]. In contrast, defensive factors include gastric mucus, prostaglandins (PGs), bicarbonate, nitric oxide (NO), growth factors, and antioxidant enzymes or antioxidant peptides, such as glutathione (GSH) [4].

All the stomach’s layers can develop a gastric ulcer. Any layer damage prevents the physiological processes in the body from functioning normally, leading to a rise in the production of stomach acid, reactive oxygen species, nitric oxide synthase, and lipid peroxidation [5]. Non-steroidal anti-inflammatory drugs (NSAIDs), alcohol intake, bacterial infection, stress, and refluxed bile salts can all cause gastric ulceration [6]. Animal models frequently employ ethanol to develop stomach ulcers as manifested by vascular damage, ulceration, and stomach cell necrosis. These side effects are the result of the body’s metabolism of ethanol, leading to the production of hydroperoxy free radicals and superoxide anions, and inducing inflammatory response by increased gastric pro-inflammatory NF-κB [5]. Because ROS have the ability to oxidize cellular proteins and lipids, they can damage the intestinal barrier and make the gut lining more permeable, which causes inflammation [7].

Proton pump inhibitors, antibiotics, H2RAs, PGs analogues, and cytoprotective medications are only a few of the pharmacological regimens that are currently available to prevent peptic ulcers and promote mucosal injury recovery [8]. The disadvantages of these drugs, however, include their ineffectiveness in treating gastrohelcosis and their substantial side effects, which include gynecomastia, hypoacidity, impotence, osteoporotic bone fractures, hypergastrinemia, and an elevated risk of cardiovascular disease [5], warranting the need to develop less harmful medications, especially over an extended period of time [9].

*Bassia indica* (Wight) A.J. Scott (Amaranthaceae) is a common wild plant through the western Mediterranean and as far as eastern Asia, including Egypt [10]. This plant is an important ethno-medicinal herb for the treatment of several ailments: tumors, inflammation, cardiotonic effects, and as an antioxidant [11,12]. The documented data revealed that *B. indica*’s different extracts are particularly rich in compounds such as flavonoids, lignans, sterols, lignanamides, coumarins, phenolic glycosides, terpenes and saponins [12]. Additionally, different extracts and isolates of the plant were reported to exhibit significant biological potentialities, such as anti-inflammatory and antimicrobial activity and cytotoxicity [13,14]. 

An important component of inflammation is mediated by high-mobility group box 1 protein (HMGB1), a chromatin binding factor that promotes inflammation. Additionally, prior studies have demonstrated that HMGB1 is one of the most prevalent damage markers that is expressed in all types of mammalian cells [15]. It was discovered that HMGB1 contributes to the development of stomach ulcers [16]. Therefore, HMGB1 represents a novel intervention factor for the control of inflammation. It acts as an extracellular signaling molecule that interacts with pattern recognition receptors such as TLR4, leading to inflammation cascade [17] that can activate NF-κB, which controls the transcription of different cytokines, such as IL-1β [18]. IL-1β markedly enhanced the phosphorylation of p38-MAPK, which has been implicated in the nuclear accumulation of Nrf2 [19]. As a result, inhibition of the HMGB1/TLR4/NF-κB signaling pathway can therefore be a potential approach for a number of illnesses mediated by HMGB1 [20].

To emphasize the utilization of *B. indica* extracts as potential sources of pharmaceutical candidates, this study is conducted to provide a more detailed overview of the anti-ulcerogenic activity of BIEE. Further, the BIEE anti-ulcerogenic effect, mediated via inhibition of the HMGB1/TLR4/NF-κB signaling and NF- κB/IL-1β/Nrf-2, were assessed as well as mitigating gut barrier disruption, and improved epithelial cell integrity, in order to ascertain that BIEE represents a potentially effective drug for treatment of stomach ulcer.

## 2. Materials and Methods

### 2.1. Plant Material Collection, Authentication and Extraction 

The collection of *B. indica* underground parts occurred during the time of plant flowering (April–May) in 2020 from the Mediterranean coast, at Baltim, Kafr Elsheikh governorate, Egypt (31°35′12.47″ N, 31°8′26.62″ E). A plant sample was authenticated and deposited with voucher number BIx80Zy/20-01916 at the herbarium of the College of Science, Mansoura University, by Prof. Ahmed M. Abdel Gawad, Professor of Plant Ecology. The collected plant materials were cleaned from soil and sand and left in a completely dry and open shady room for drying at room temperature. After complete drying, the plant material was crushed into powder via a clean plant grindery. 

The powdered plant material (370 g) was extracted using maceration in 70% EtOH (4 L) at 25 °C (±3) for one week and then filtered. The extraction process was performed three times. Overall afforded extract was collected in liquor form and completely dried under reduced pressure to afford a dark black gum (14.2 g). The obtained extract (completely free of EtOH) was then stored at 4 °C in the refrigerator until further biological evaluation.

### 2.2. Animals

At the National Research Centre’s animal house colony in Giza, Egypt, adult male Wistar albino rats (weight: 200–220 g) were obtained. All animals were kept in well-ventilated metal cages at a temperature of 22 °C, 55 °F, and 12 h of darkness and light cycles. They were given unlimited amounts of water along with standard rat meal pellets containing 21% proteins, 3.48% fats, 3.71% raw fibre, and 1% multivitamins. Ingredients include yellow maize, soybean meal (44%) and corn gluten (60%), as well as limestone, hulled sunflower cake, crude soybean oil, a combination of vitamins and minerals, methionine, and an anti-fungicide. Water was available at all times during the experiment. The guidelines of the National Research Centre’s Ethics Committee (registration number 4411022023) were followed in the implementation of the study’s protocol and procedures. 

### 2.3. Drugs and Chemicals

Omeprazole was purchased from Sigma-Aldrich (St. Louis, MO, USA). Ethanol (96%) was purchased from Merck Millipore (Burlington, MA, USA).

### 2.4. Experimental Design

Thirty-six healthy male rats were weighed and given enough water and food to adapt to the environment, kept under 12 h at light and 12 h in darkness. After adaptation, rats were randomly grouped into 6 groups (6 rats each). Rats were given enough water and food with free access. All groups, with the exception of the first, received the medicines via gavage. The rats were divided into the following groups [21].

The 1st group (normal control): Rats were administrated only normal saline (5 mL/kg) orally via intragastric gavage; 2nd group (negative ulcer control): Rats were administrated ethanol (EtOH; 99.9%) at a dose of 5 mL/kg orally via intragastric gavage [22]; 3rd group (Reference drug): Rats were administrated Omeprazole (20 mg/kg dissolved in bi-distilled water) orally via intragastric gavage [22]; 4th, 5th and 6th groups (pre-treatment groups): rats were administrated BIEE at doses of 25, 50 and 100 mg/kg, respectively, orally via intragastric gavage. The oral treatment of all the rats of the 3rd, 4th, 5th, and 6th groups were performed seven days prior to ethanol oral induction. The first and second groups received sterile saline by intra-gastric gavage during the gavage of the medicines in the 3rd to 6th groups, simulating the stress circumstances brought on by the gavage.

Animals were sedated with a cotton ball saturated with diethyl ether for 2–5 min in a desiccator after 1 h of ulcer induction, and were then put to death by cervical dislocation. Animal stomachs were swiftly removed, cracked along the greater curve, and their contents extracted. After gently rinsing with cold phosphate-buffered saline solution to eliminate any blood clots, the gastric tissue samples were inspected macroscopically to determine the gastric ulcer index. The stomach was then dried between two filter papers and divided into three portions, one of which was used to make 10% homogenate by homogenizing it in ice-cold saline to assess antioxidant properties and indicators of oxidative stress, and this was frozen at 20 °C. In order to conduct a subsequent Western blot analysis, the second portion was kept at 80 °C. The third part was then preserved in 10% formalin for histological analysis.

### 2.5. Index of Gastric Ulcers and Percentage Inhibition

According to the procedure recommended by [23], the ulcer inhibition percentage (I%) and ulcer index (UI) in units of square millimeters (mm^2^) were calculated, with a slight change. In this procedure, the wounded area’s surface was first measured with a ruler, and the ulcer’s severity was calculated depending on the ulcer’s degree. Utilizing the following formulas, the ulcer index (UI) and ulcer inhibition percent (I%) were calculated [24]:

$$\mathrm{Ulcer}\;\mathrm{index}\;\left(\mathrm{UI}\right)=\frac{\mathrm{Totally}\;\mathrm{ulcer}\;\mathrm{score}}{\mathrm{Ulcerated}\;\mathrm{rats}\;\mathrm{Number}}$$

$$\mathrm{Ulcer}\;\mathrm{Inhibition}\;\mathrm{Percent}\;\left(I\%\right)=\frac{\mathrm{UI}\left(\mathrm{Control}\right)-\mathrm{UI}\left(\mathrm{pretreated}\right)}{\mathrm{UI}\left(\mathrm{Control}\right)}\times 100$$

### 2.6. Histological Examination

Different sections from the stomach tissues of all groups were cut and fixed in 10% buffered formalin. The tissues underwent standard processing before being embedded in paraffin wax. Next, gastric tissues were cut into 5 µm thick sections and stained with H&E. The gastric mucosal and submucosal damage was assessed in 10 random high-power fields (40×), as described by [25]. Epithelial cell loss (score: 0–3), hemorrhage (score: 0–4), and inflammatory cellular infiltrates (score: 0–3) were the pathological parameters used for the assessment of the gastric damage. The total pathologic score is the sum of these three partial scores.

### 2.7. Immunohistochemical Analysis

The immunohistochemical technique for the demonstration of caspase-3 expression in the gastric glandular tissues was performed. Initially, alcohol was used to dewax and rehydrate the paraffin-embedded gastric tissue sections. The endogenous peroxidase activity was then inhibited by incubating the sections in 3% hydrogen peroxide. Following that, tissues were incubated with rabbit monoclonal anti-caspase-3 (EPR 18297) (ab 184787) (abcam) and rabbit polyclonal anti-TLR4, 1:50, Santa Cruz, CA, USA. The immune reactivity was visualized using diaminobenzidine (DAB). Based on the percentage of positively stained cells, the immunological response was semi-quantitatively evaluated in 10 randomly selected high-power fields (40×). The results were graded on a scale of 0 to 3, with 0 denoting no staining, 1 denoting positive staining in 30% of cells or HPF, 2 denoting positive staining in 30% to 70% of cells or HPF, and 3 denoting positive staining in more than 70% of cells or HPF.

### 2.8. Biochemical Evaluation (Production of a Stomach Homogenate)

Animals were sacrificed, tissues were properly cleaned, and rinsed with ice to measure biochemical data. Between the folds of filter paper, they were gently blotted before being weighed in an analytical balance. A polytron homogenizer was used to prepare 10% of homogenate at 40 °C in 0.05 M phosphate buffer (pH 7). For the purpose of eliminating cell debris, unbroken cells, nuclei, erythrocytes, and mitochondria, the homogenate was centrifuged at 10,000 rpm for 20 min. In accordance with the recommendations of the manufacturer, the supernatant (cytoplasmic extract) was employed to evaluate biochemical parameters.

### 2.9. Estimation of MDA and GSH Contents

Bio Diagnostic Company kits were utilized for the enzymatic colorimetric measurement of MDA and GSH at wave length 534 nm in accordance with the method of [26] for MDA, and 405 nm according to the method of [27] for GSH.

### 2.10. Western Blot Assay of NF-κB, HMGB1

#### 2.10.1. Protein Extraction Procedure

Each homogenized tissue sample was treated with the Ready PrepTM protein extraction kit (total protein) supplied by Bio-Rad Inc. (Catalogue #163-2086) (Bio-Rad Laboratories, Inc. is an American Manufacturer, Hercules, CA, USA) in accordance with the manufacturer’s instructions. Bio Basic Inc. (Markham, ON, Canada) provides the Bradford Protein Assay Kit (SK3041) for quantitative protein analysis. In order to calculate the protein content in each sample, a Bradford assay was carried out in accordance with the manufacturer’s instructions. Then, an equal volume of 2× Laemmli sample buffer containing 4% SDS, 10% 2-mercaptoethanol, 20% glycerol, 0.004% bromophenol blue, and 0.125 M Tris HCl was loaded onto each sample’s 20 g protein concentration. When the pH was measured, it was raised to 6.8. To guarantee that the protein was denaturated before loading on the polyacrylamide gel electrophoresis, each combination was cooked at 95 °C for 5 min.

#### 2.10.2. Protein Separation by Electrophoresis 

SDS-PAGE, i.e., Sodium Dodecyl Sulphate Poly Acrylamide Gel Electrophoresis—a common method for separating proteins based on their molecular weight—was used to separate the samples on a polyacrylamide gel. The TGX Stain-Free^TM^ Fast Cast^TM^ Acrylamide Kit (SDS-PAGE), supplied by Bio-Rad Laboratories Inc. Cat #161-0181, was used to create polyacrylamide gels. The manufacturer’s instructions were followed while preparing the SDS-PAGE TGX Stain-Free Fast Cast.

#### 2.10.3. Transferring Proteins from a Gel to a Membrane (Protein Blotting)

The gel was put together in a transfer sandwich with a PVDF membrane, the gel, and the filter paper, from bottom to top. The sandwich was added to the transfer tank along with the 1× transfer buffer, which contains 25 mM Tris, 190 mM Glycine, and 20% methanol. Afterward, the blot was run on the BioRad Trans-Blot (Santa Cruz Biotechnology, Inc., Santa Cruz, CA, USA) Turbo for 7 min at 25 V to allow protein bands to transfer from gel to membrane. The membrane was blocked for 1 h at room temperature in tris-buffered saline with Tween 20 (TBST) buffer and 3% bovine serum albumin (BSA). In addition, 20 mM Tris pH 7.5, 150 mM NaCl, 0.1% Tween 20, and 3% bovine serum albumin (BSA) made up the blocking buffer. In TBST, NF-B and HMGB1 primary antibodies were diluted in accordance with the manufacturer’s (www.Scbt.com, accessed on 4 November 2022) recommendations. Each primary antibody solution was incubated at 4 °C overnight with the blotted target protein. The blot was rinsed with TBST 3–5 times for 5 min. The blotted target protein was incubated for 1 h at room temperature in the HRP-conjugated secondary antibody (Goat anti-rabbit IgG- HRP-1 mg Goat mab–Novus Biologicals) solution. 

#### 2.10.4. Quantitative Data Analysis and Imaging

According to the manufacturer’s protocol, the chemiluminescent substrate (Clarity^TM^ Western ECL substrate, Bio-Rad cat#170-5060) was applied to the blot. Briefly, equal quantities of solution B (peroxidase solution) and solution A (Clarity Western luminal/enhancer solution) were added. A CCD camera-based imager was used to record the chemiluminescent signals. By protein normalization on the Chemi Doc MP imager, image analysis software was used to read the band intensity of the target proteins against the control sample beta actin (housekeeping protein) [28].

### 2.11. Estimation of IL-1β and Nrf2 Contents

Stomach IL-1β and Nrf2 contents were assessed using ELISA kits from Elabscience Biotechnology Co., Ltd., Houston, TX, USA and were expressed as pg/mL. The manufacturer’s instructions were followed for each step in the utilized kits’ processes.

### 2.12. High-Resolution Ultra-Performance Liquid Chromatography-Mass Spectrometry Analysis (UPLC-ESI–Qtof-MS)

One gram of the powdered *B. indica* was extracted for one hour over an ultrasonic bath (Branson Ultrasonic Corporation, Danbury, CT, USA) with a solution of 70% hydroethanol, filtered, and centrifuged for 15 min. The extract liquid’s clear supernatant was then removed and subjected to UPLC-ESI-Qtof-MS analysis. The identical procedure and circumstances used in the prior investigation were followed for the UPLC-ESI-Qtof-MS analysis [29,30]. In brief, 10 mg of the dried finely pulverised plant sample was extracted through the addition of 100% MeOH (2 mL), containing umbelliferone (10 g/mL^−1^) as an internal standard, with sonication and frequent shaking for 20 min. The debris was removed by centrifuging for 10 min at 12,000× *g*. Then, the filtered extract (22-μm) was treated to solid-phase extraction using a C18 cartridge. Then 2 μL of the plant extract was loaded on an HSS T3 column (100 × 1.0 mm, particle size 1.8 μm; Waters) installed on an ACQUITY UPLC system (Waters, Milford, MA, USA) equipped with a 6540 Ultra-High-Definition (UHD) Accurate-Mass Q-TOFLC/MS (Agilent, Palo Alto, CA, USA) coupled to an ESI interface and operated in positive or negative ion mode. The metabolites were characterized by generating a possible formula with a mass accuracy limit of 10 ppm, also taking into account RT, tandem MS2 data, and examining reference literature and the Phytochemical Dictionary of Natural Products Database. Peaks were noted in negative as well as positive ion modes (deviating values are shown in brackets).

### 2.13. Statistical Analysis

Graph Pad Prism was used to conduct a variance analysis on the data. Standard Error of the Mean (SEM) was used to present the results. The means were compared using one-way analysis of variance (ANOVA), and then Tukey’s multiple comparison tests were utilized. At *p* < 0.05, differences in means were considered significant. 

## 3. Results

### 3.1. Effect of B. indica EtOH Extract on Ulcer Index

**Figure 1:**
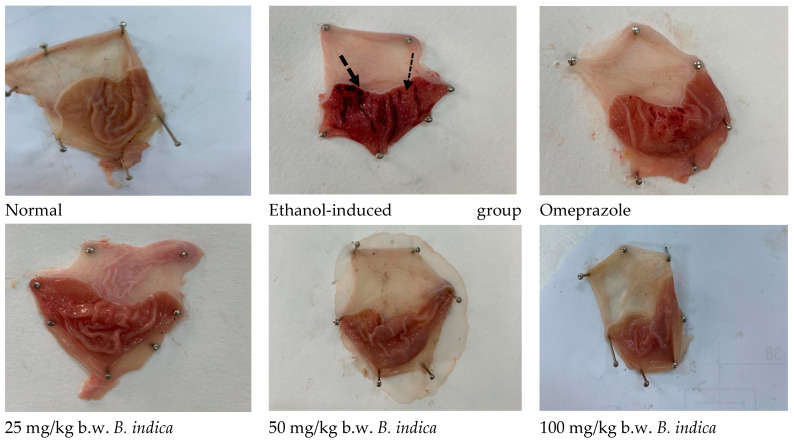
Effect of *B. indica* EtOH extract on ethanol-induced gastric mucosal injury in rats. The normal group had no macroscopic lesions. The control group’s stomach mucosa displayed severe ethanol-induced lesions in the form of hemorrhagic bands. Pre-treatment with omeprazole (20 mg/kg) or *B. indica* EtOH extract (25, 50, 100 mg/kg) considerably decreased lesions of gastric mucosa hemorrhage, respectively.

**Figure 2:**
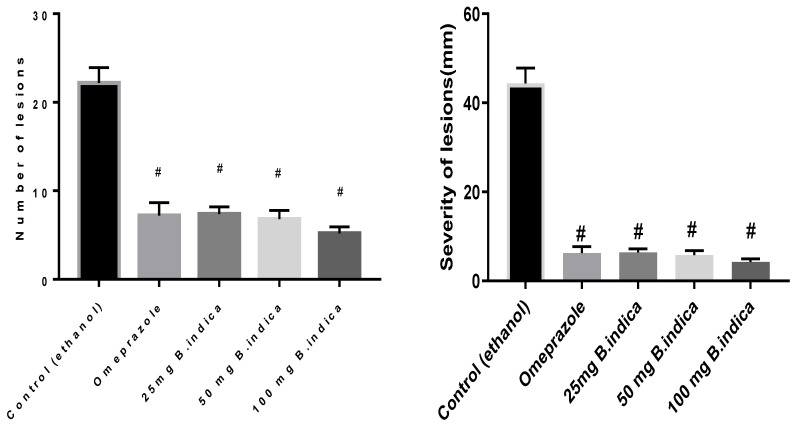
Effect of *B. indica* EtOH extract on ethanol induced gastric lesions number and severity. Each bar represents the mean ± SE of 6 rats. # denoted a significant difference from the control (ethanol) group at *p* < 0.05, employing the Tukey-Kramer multiple comparisons test after one-way ANOVA.

The impact of BIEE on the frequency and length of stomach lesions (Figure 1) brought on by ethanol was calculated (Figure 2). In the normal group, there was no macroscopic damage. In contrast, the ethanol diseased group showed severe gastric mucosal injuries as manifested by hyperemia and linear bleedings (Figure 1), numbering 22.2 ± 0.71, and severity of 44.40 ± 1.4. Pre-treatment with BIEE at three dose levels of 25, 50, 100 mg showed significant lower levels at 66.7%, 69.4%, 76.5%, and 85.5%, 86.9%, 90.36% for number and severity, respectively, as compared to the control group. In the same situation, pre-treatment with omeprazole dramatically reduced both number and severity, as compared to the control group, by 67.5% and 86%, respectively (Figure 2).

### 3.2. Effect of B. indica EtOH Extract on MDA and GSH Levels

**Figure 3:**
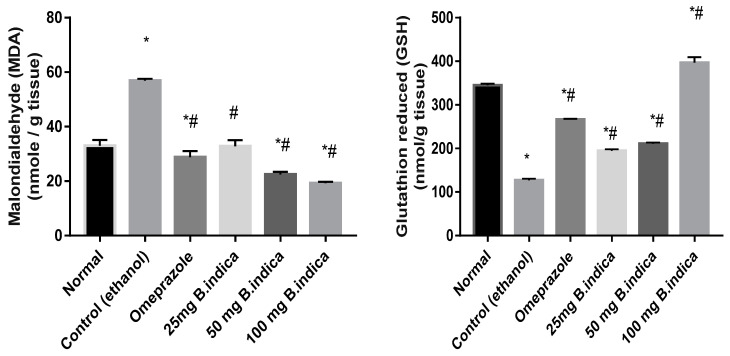
Effect of *B. indica* EtOH extract on MDA and GSH contents in ethanol induced gastric lesions. Each bar represents the mean ± SE of 6 rats. Significant difference from the normal group indicated by * at *p* < 0.05. # denoted a significant difference from the control (ethanol) group at *p* < 0.05, employing the Tukey-Kramer multiple comparisons test after one-way ANOVA.

Administration of ethanol significantly increased MDA concurrent with a decrease in GSH levels by 1.7 and 2.7 fold, respectively, as compared with the normal group. Pre-treatment with BIEE at the three doses of 25, 50 and 100 mg/kg b.w, in contrast, significantly decreased MDA level, concurrent with increase in GSH by 42.4%, 60.4%, 64.75% and 53.3%, 65.8%, 211.6%, respectively, as compared with the control group. Likewise, pre-treatment with omeprazole significantly decreased contents of MDA and increased that of GSH by 49.3% and 110%, respectively, as compared with the control group (Figure 3).

### 3.3. Effect of B. indica EtOH Extract on Protein Expression of HMGB1 and NF-κB 

**Figure 4:**
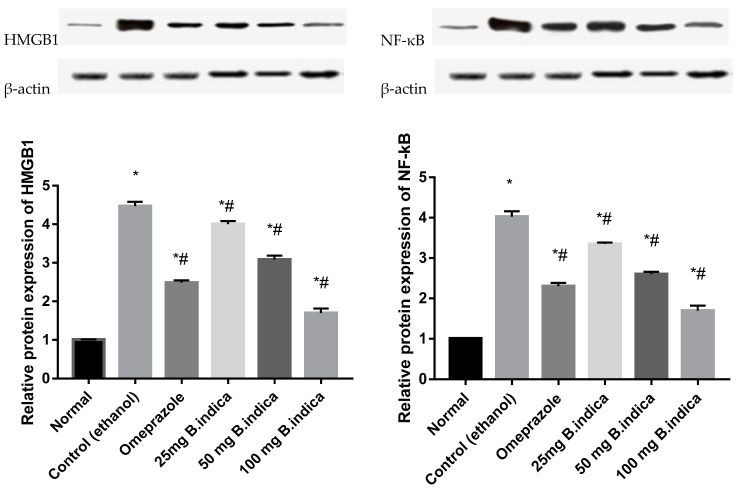
The effect of *B. indica* EtOH extract on protein expression of HMGB1 and NF-κB in ethanol-induced gastric ulcer in rats. Each bar represents the mean ± SE of 6 rats. Significant difference from the normal group indicated by * at *p* < 0.05. # denoted a significant difference from the control (ethanol) group at *p* < 0.05, employing the Tukey-Kramer multiple comparisons test after one-way ANOVA.

In comparison to the healthy normal group, administration of ethanol significantly increased the protein expression of HMGB1 and NF-B by 4.4 and 4 fold, respectively, whereas pre-treatment with BIEE at the three doses of 25, 50 and 100 mg/kg b.w significantly decreased protein expression of HMGB1 and NF-κB by 10.3%, 31%, 62%, and 16.8%, 35.4%, 57.9%, respectively, compared with control group. When compared to the control group, pre-treatment with omeprazole significantly reduced the expression of the HMGB1 and NF-B proteins by 44.5% and 42.7%, respectively (Figure 4).

### 3.4. Effect of B. indica EtOH Extract on IL-1β and Nuclear Nrf-2

**Figure 5:**
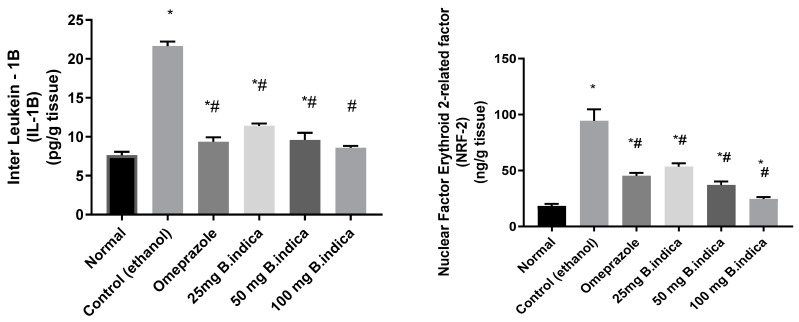
Effect of *B. indica* on IL-1β and nuclear Nrf-2 contents in ethanol induced gastric lesions. Each bar represents the mean ± SE of 6 rats. Significant difference from the normal group indicated by * at *p* < 0.05. # denoted a significant difference from the control (ethanol) group at *p* < 0.05, employing the Tukey-Kramer multiple comparisons test after one-way ANOVA.

Administration of ethanol significantly increased IL-1β, and Nrf-2 levels by 2.8 and 5.2 fold, respectively, as compared to the normal group, whereas pre-treatment with BIEE at three doses of 25, 50 and 100 mg/kg b.w significantly decreased contents of IL-1β and Nrf-2 by 47.2%, 55.6%, 60.6%, and 44.6%, 61.86%, 74.5%, respectively, compared with the control group. Pre-treatment with omeprazole as positive drug control significantly decreased IL-1β, and Nrf-2 contents by 56.9% and 52.96%, respectively, as compared with the control group (Figure 5).

### 3.5. Effect of B. indica EtOH Extract on Stomach Morphological Changes induced by EtOH

**Figure 6:**
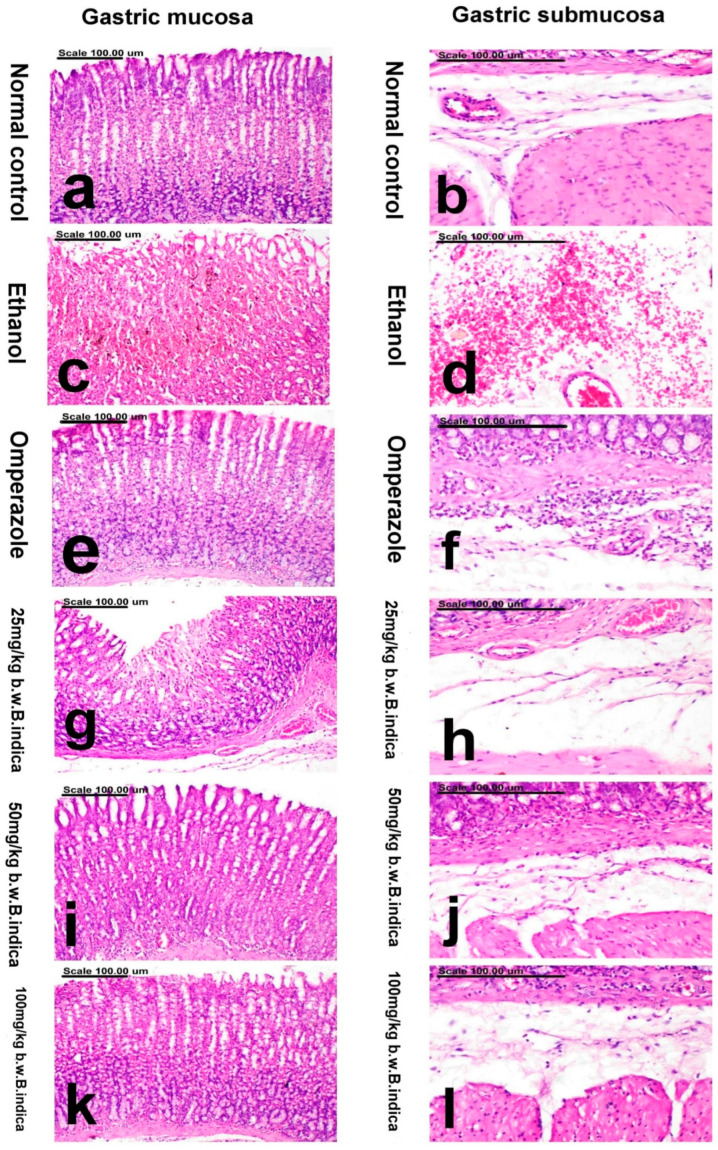
Photomicrograph of the gastric mucosa and submucosa represents the following: (**a**,**b**) normal group showing normal mucosal epithelium and normal tubular glands (**a**) and normal submucosa (**b**), (**c**,**d**) ethhanol group showing extensive necrosis of mucosal epithelium and the gastric glands, concurrently with massive mucosal (**c**) and submucosal hemorrhage (**d**), (**e**,**f**) Omeprazole group showing normal gastric mucosa (**e**) and few leucocytic infiltrates in the submucosa (**f**), (**g**,**h**) *B. indica* EtOH extract (25 mg/kg b.w) showing focal necrosis of the gastric mucosa (**g**) and normal submucosa (**h**), (**i**,**j**) *B. indica* EtOH extract (50 mg/kg b.w) showing restoration of the gastric mucosa (**i**) and normal submucosa (**j**), (**k**,**l**) *B. indica* EtOH extract (100 mg/kg b.w) showing normal gastric mucosa (**k**) and submucosa (**l**). (Stain: H&E; Scale bar = 100 µm).

The overall pathologic score of gastric injury recorded in all groups is shown in Table 1. The stomach of normal control rats revealed normal histological structure, with normal mucosal epithelium and normal tubular glands, in addition to normal submucosa (Figure 6a,b). Meanwhile, stomach in the ethanol group revealed diffuse ulcerative lesions with extensive necrosis of mucosal epithelium and the gastric glands, concurrently with massive mucosal and submucosal hemorrhage (Figure 6c,d). Significant amelioration with a decrease in the pathologic score was recorded in the omeprazole group and other treated groups. Reparative effect, with regeneration of the gastric mucosa and only a few leucocytic infiltrates in the submucosa, was demonstrated in the omeprazole group (Figure 6e,f). Similarly, marked improvement was demonstrated in BIEE, particularly in the medium and high dose groups. Only focal necrosis of the gastric mucosal epithelium and tubular glands, in addition to normal submucosa, were demonstrated in the low-dose group (Figure 6g,h). Restoration of the gastric mucosa with minute focal hemorrhage and normal submucosa were demonstrated in the medium dose group (Figure 6i,j). Normal gastric mucosa and submucosa, with sparse necrosis of the superficial epithelium, were demonstrated in the high-dose group (Figure 6k,l).

### 3.6. Effect of B. indica EtOH Extract on TLR4 and Caspase-3 Immunohistochemical Expression

**Figure 7:**
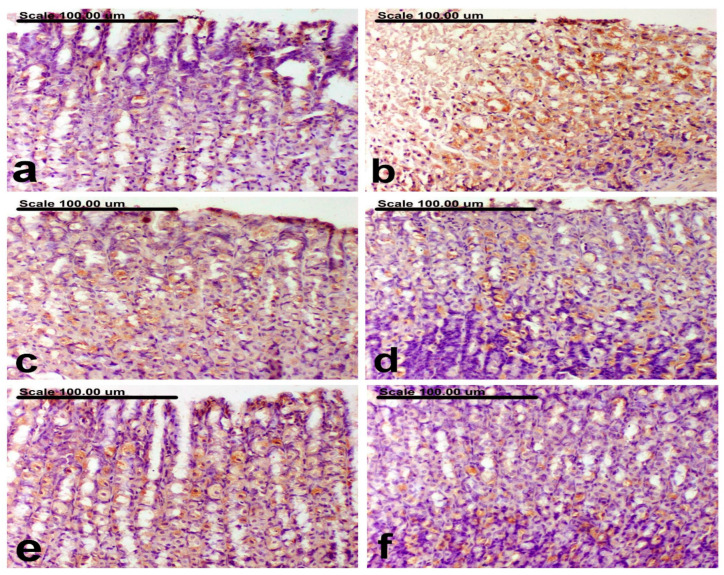
Photomicrograph of the gastric mucosa immunohistochemically stained with anti-TLR4 antibody represents the following: (**a**) normal group showing sparse TLR4 and Caspase-3-positively stained cells with weak brown cytoplasmic staining, (**b**) ethhanol group showing increased % of TLR4 positively stained cells, with strong brown cytoplasmic staining, (**c**) Omeprazole ombrazole group showing pronounced decrease of TLR4-positively stained cells, (**d**) *B. indica* EtOH extract (25 mg/kg b.w) showing decrease of TLR4-positively stained cells, (**e**) *B. indica* EtOH extract (50 mg/kg b.w) showing decrease of TLR4-positively stained cells, with moderate cytoplasmic staining, (**f**) *B. indica* EtOH extract (100 mg/kg b.w) showing significant decrease of TLR4-positively stained cells, with weak cytoplasmic staining. (TLR4 immunohistochemical staining; Scale bar = 100 µm).

**Figure 8:**
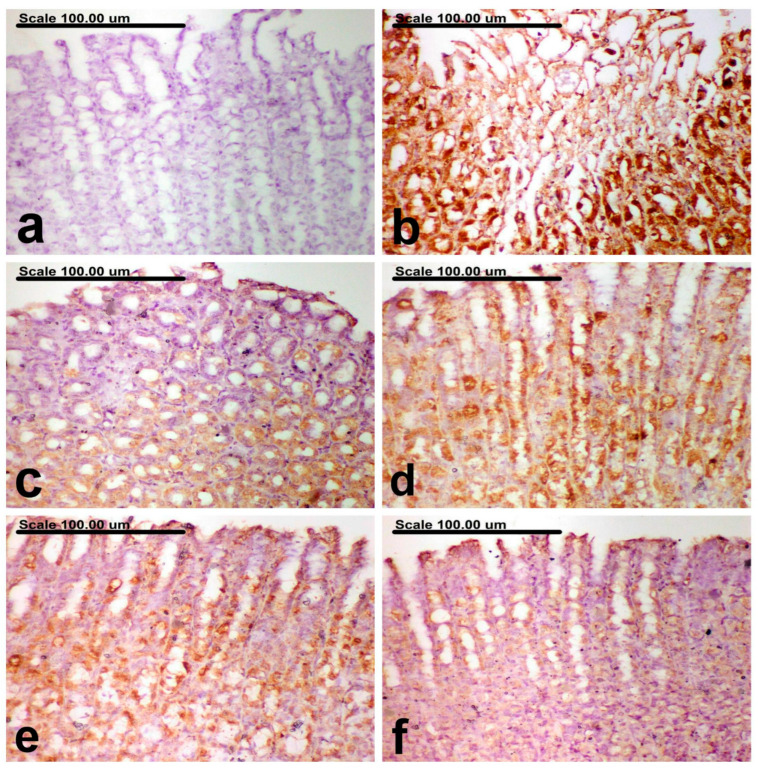
Photomicrograph of the gastric mucosa immunohistochemically stained with anti-Caspase-3 antibody represents the following: (**a**) normal group showing no Caspase-3-positively stained cells, (**b**) ethhanol group showing increased % of Caspase-3-positively stained cells, with diffuse strong brown cytoplasmic and/or staining, (**c**) Omeprazole group showing decrease of Caspase-3-positively stained cells, with weak brown cytoplasmic staining, (**d**) *B. indica* EtOH extract (25 mg/kg b.w) showing decrease of Caspase-3-positively stained cells, (**e**) *B. indica* EtOH extract (50 mg/kg b.w) showing decrease of Caspase-3-positively stained cells, with moderate cytoplasmic staining, (**f**) *B. indica* EtOH extract (100 mg/kg b.w) showing significant decrease of Caspase-3-positively stained cells, with faint weak cytoplasmic staining. (Caspase-3 immunohistochemical staining; Scale bar = 100 µm).

The outcomes of TLR4 and Caspase-3 expression found in the stomach tissues of untreated groups and normal groups are displayed in Table 2. Immunohistochemical analysis of the gastric tissues of the normal control rats revealed sparse TLR4- and caspase-3-positively stained cells with weak brown cytoplasmic staining (Figure 7a and Figure 8a, respectively). On the contrary, increased expression levels of TLR4 and Caspase-3, along with an increased percentage of positively stained cells with strong brown cytoplasmic staining, were recorded in the ethanol group (Figure 7b and Figure 8b, respectively). Amelioration was recorded in the omeprazole group, with a pronounced decrease of TLR4 and caspase-3 expression and a reduction of the percentage of positively stained cells (Figure 7c and Figure 8c, respectively). In comparison with the ethanol group, a significant reduction of TLR4 and Caspase-3 expression was recorded in BIEE. A non-significant difference was recorded between the low and medium dose groups, both showing a pronounced decrease of TLR4 and caspase-3-positively stained cells (Figure 7d and Figure 8e for TLR4 and Figure 8d,e for caspase-3), although a significant decrease of TLR4 and caspase-3-positively stained cells was recorded in the high dose group (Figure 7f and Figure 8f, respectively).

### 3.7. Metabolites Profiling of B. indica EtOH Extract via UPLC-ESI–Qtof-MS

**Figure 9:**
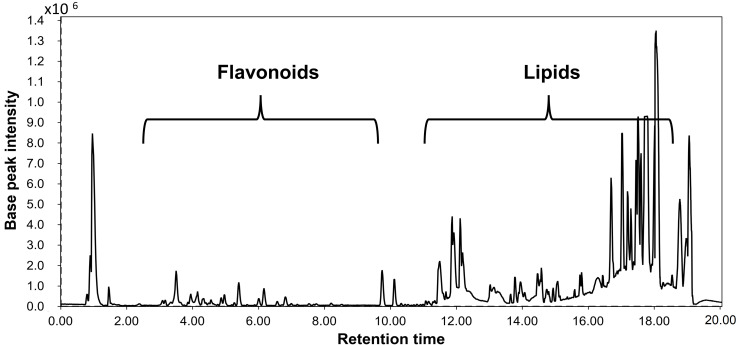
Representative UHPLC–MS traces analyzed in the positive ion mode of *Bassia indica* EtOH extract showing two main classes, *viz*., flavonoids and lipids.

In the present study, UPLC-ESI–Qtof-MS analysis has allowed for the comprehensive characterization of the BIEE metabolites via an untargeted approach (Figure 9). In total, 40 metabolites were annotated mostly belonged to two main chemical classes, *viz*., flavonoids and lipids, in line with the reported literature [31]. The identification strategy of the detected metabolites was based on their retention times, experimental *m*/*z*, molecular formulas, mass errors and their MS^2^ fragments, as shown in (Table 3). 

#### 3.7.1. Identification of Flavonoids

Fifteen free, glycosylated and/or acylated flavonoids were identified in BIEE, based on their MS/MS fragmentation pattern (Table 3) and in agreement with the reported literature [12]. In detail, five quercetin glycosides were annotated based on their fragment ions at *m*/*z* 303 [M + H]^+^ typical for the quercetin aglycon, in addition to the characteristic MS/MS neutral losses of their sugar residues, e.g., hexose (−162 Da), pentose (−132 Da) and rhamnose (−146 Da). For example, peak 5 at *m*/*z* 773.2123 [M + H]^+^ was annotated as quercetin-*O*-hexosyl-rhamnosyl-hexoside supported by fragment peaks at *m*/*z* 611 [M + H−162] ^+^ for the loss of the hexosyl moiety, *m*/*z* 465 [M + H−162−146] ^+^ for further loss of the rhamnosyl moiety and *m*/*z* 303 for the loss of the other hexosyl moiety [M + H−162−146−162]^+^ (Figure S1). In addition, the identification of quercetin-*O*-pentosyl-hexoside (peak 10), quercetin-*O*-rhamnosyl-hexoside (peak 11) and quercetin *O*-hexoside (peak 12) was also facilitated based on the abundant aglycon product ions at *m*/*z* 303 due to the successive loss of the pentosyl-hexoside, rhamnosyl-hexoside and hexosyl moieties, respectively (Table 3). Such neutral losses of the sugar residues typify the homolytic cleavage of their *O*-glycosidic bonds and confirm the *O*-type linkage [32]. Likewise, a similar fragmentation pattern was observed for kaempferol and its glycosides, e.g., peaks 8, 13 and 16, corresponded to kaempferol-*O*-rhamnosyl-di-hexoside (Figure S2), kaempferol-*O*-hexosyl-rhamnoside and kaempferol-*O*-hexoside (Figure S3), respectively, also showing the typical neutral losses of their sugar residues with characteristic product ions at *m*/*z* 287, typical for the kaempferol aglycon fragment (Table 3). Both quercetin and kaempferol are flavonols known for their anti-inflammatory and antioxidant effects in in vitro studies [33] Moreover, several reports showed the potential of quercetin ameliorating activity against indomethacin-induced gastric ulcers in rats, owing to its anti-apoptotic effect [34]. 

Methoxy flavonoids such as isorhamnetin and its glycosides have also been identified in the BIEE in agreement with the reported literature [35]. For example, peak 6 at *m*/*z* 787.2292 [M + H]^+^ was assigned as isorhamnetin-*O*-rhamnosyl-di-hexoside based on the successive loss of the rhamnosyl and the two hexosyl moieties at *m*/*z* 641, 479 and 317, respectively (Figure S4), yielding the aglycon fragment of isorhamnetin (Table 3). Likewise, peak 14 displayed [M + H]^+^ at *m*/*z* 625.1757, annotated as isorhamnetin-*O*-rhamnosyl-hexoside, with MS^2^ fragment ions at *m*/*z* 479 [M + H-146]^+^ and *m*/*z* 317 [M + H−146−162]^+^ corresponding to the neutral loss of the sugar residues (Figure S5). Isorhamnetin is known for its potent antioxidant and anti-inflammatory properties mainly via suppressing the formation of cytokines and infiltration of inflammatory cells, in addition to inhibiting p38 and NF-κB pathways, thus its role in alleviation of GIT mucosal injuries has been well-documented [36]. Acylated flavonoids also have been characterized in the BIEE in agreement with the reported literature [35]. In this context, peak 4 was identified as cyanidin-*O*-hexosyl coumaryl-trihexoside, supported by its product ions at *m*/*z* 757, 595, 449, and 287, corresponding to the neutral loss of the sugar and coumaroyl residues (Table 3).

#### 3.7.2. Identification of Lipids

Next to flavonoids, lipids amounted to the second abundant class in BIEE predominated by hydroxylated and nitrogenous forms (Table 3). In details, peaks 23 (*m*/*z* 295.2261, C_18_H_31_O_3_^+^) and 34 (*m*/*z* 299.2573, C_18_H_35_O_3_^+^) were identified as mono-hydroxylated forms of octadecatrienoic acid and octadecenoic acid, respectively. Both peaks displayed MS^2^ fragments of (−18 Da) from their parent ion peaks corresponded to the loss of water molecule and indicative for the extra hydroxyl group. Similarly, peaks 20 (*m*/*z* 347.2426, C_18_H_35_O_6_^+^) displayed MS^2^ fragments at *m*/*z* 311 [M + H-36]^+^ corresponding to a loss of two water molecules, and were thus annotated as dihydroxy-octade-canedioic acid.

Several peaks with even mass weights have also been characterized in BIEE, suggesting the presence of a nitrogen atom in their structure, such as peaks 21 (*m*/*z* 246.2423, C_14_H_32_NO_2_^+^), 24 (*m*/*z* 274.2735, C_16_H_36_NO_2_^+^), 25 (*m*/*z* 318.2996, C_18_H_40_NO_3_^+^) and 27 (*m*/*z* 302.3046, C_18_H_40_NO_2_^+^), identified as tetra-decasphinganine, hexa-decasphinganine, phsphingosine and sphinganine, respectively (Table 3). Additionally, several fatty acid amides have been also identified, also based on their even mass weights, such as peaks 32 (*m*/*z* 284.2948, C_18_H_38_NO^+^), 36 (*m*/*z* 254.248, C_16_H_32_NO^+^) and 38 (*m*/*z* 310.3097, C_20_H_40_NO^+^), annotated as octadecanamide, hexadecenamide and eicosenamide, respectively. 

#### 3.7.3. Identification of Amino Acids and Triterpenes

Three amino acids were also identified in the BIEE, including valine (*m*/*z* 118.0864, peak 1), glutamyl-glycine (*m*/*z* 205.082, peak 2) and fructosyl phenylalanine (*m*/*z* 328.1379, peak 3). Fructosyl phenylalanine showed characteristic MS^2^ fragments at *m*/*z* 310 (−17 Da), corresponding to the loss of the terminal amino moiety, and *m*/*z* 166 corresponded to the phenylalanine fragment, confirming its structure. 

Bassic acid, a pentacyclic triterpenoidal saponin previously reported in the *Bassia* genus [37], has also been characterized in this study (Table 3). Bassic acid, peak 26, has been identified based on its [M + H]^+^ at *m*/*z* (487.3414, C_30_H_47_O_5_^+^) with MS^2^ fragment ions at *m*/*z* 469 [M + H−18]^+^ corresponding to a loss of water molecule, and *m*/*z* 441 [M + H−18−28]^+^ corresponding to a further loss of CO moiety.

## 4. Discussion

Although the cause of peptic ulcers is unknown, they are most often thought to be caused by an imbalance between protective and aggregative factors, which are largely influenced by lifestyle choices, such as a sedentary lifestyle, eating spicy food, drinking alcohol, using drugs, and contracting various bacterial infections, such as *Helicobacter pylori* [38]. 

As part of this investigation, rats given ethanol displayed mucosal lesions and edema that were relatively more extensive, in accordance with [39], confirming that ethanol as a necrotizing agent readily penetrating the gastric mucosa and producing gastric lesions by vascular damage exerts direct toxic effect on the epithelium due to neutrophil infiltration in the ulcerated gastric tissue. As opposed to omeprazole, which is the conventional medication, BIEE pre-treatment for 7 days considerably reduced stomach damage to a lesser amount and accelerated gastric recovery. Additionally, lessened histopathological changes and an influx of leucocytes were found, both of which pointed to the substance’s anti-ulcer properties. 

Ethanol is linked to the purine breakdown process that leads to excessive ROS generation, mediating oxidative damage of lipid peroxidation, cell death, and epithelial damage [40]. Extravasation of neutrophils to the site of damage plays a crucial part in the development of gastric mucosa damage and inflammation [41].

Oxidative stress in ethanol-treated rats was demonstrated by a considerable increase in MDA level together with a decrease in GSH level and in agreement with [42]. Indicating its antioxidant activity, the BIEE pre-treatment retained GSH and reduced stomach MDA contents.

Western blotting of ethanol treated rats revealed an increase in HMGB1 protein, as reported by [22], demonstrating that HMGB1 plays a key role in the repair of stomach ulcers. HMGB1 is normally located in the nucleus and binds to chromatin, to actively and passively shuttle from the nucleus to the cytoplasm under elevated reactive oxygen species (ROS) and then into the extracellular space, where it exerts its proinflammatory activity as it functions as damage-associated molecular pattern molecules (DAMPs), mediating inflammation and immune responses acting through TLR-4 [43,44]. The pre-treatment with BIEE reduced the level of HMGB1 expression, which plays a part in accelerating the healing of stomach ulcers, These results are in agreement with previous findings of Manivannan, et al. [45], on the inflammatory responses caused by HMGB1, posing it as a functional biomarker for brain injury and neuro-inflammation and, likewise, as potential target in disease management to be explored further in the case of ulcer.

Immunohistochemistry and Western blot analyses of ethanol treated rats showed significant increase in TLR-4, NF- κB protein and proinflammatory cytokine IL-1β in agreement with [46]. HMGB1 can stimulate cytokines production via Toll-like receptor 4 (TLR4), which causes inflammation and draws leucocytes to the site of tissue damage, causing significant in vivo inflammation [47]. The activation of NF-B may result from this binding to TLR4, which translocate from the cytoplasm to the nucleus, binding to DNA to regulate transcription of various cytokines [48], including IL-1β, which is associated with a degree of ulceration, as IL-1B is an important contributing factor in intestinal mucosal injury [49]. Pre-treatment with BIEE effectively inhibited the increase of TLR-4, reduction of IL-1β expression via suppressing of NF-κB. Downregulation of TLR4 plays a vital role in gastric ulcer healing [50]. Based on various studies, anti-inflammatory action is crucial for preventing peptic ulcers [51]. Meng, et al. [52], demonstrated that decreasing the activity of NF-κB and decreased levels of pro-inflammatory cytokine IL-1β relieve ethanol-induced gastric ulcer. 

Ethanol treated rats showed significant increase in nuclear Nrf2 [53], attributed to an increase in IL-1β that markedly enhanced the phosphorylation of p38-MAPK, known to be implicated in the nuclear accumulation of Nrf2 [19]. Pre-treatment with BIEE effectively inhibited increased Nrf2 level.

Throughout the history of science, natural products have shown their influence on gastric ulcers, with both protective and curative effect [54]. In our current study, UPLC-ESI–Qtof-MS analysis revealed the identification of two major metabolite classes, flavonoids and lipids, along with a minor appearance of triterpenoids. These metabolites have formerly proved their potency as cytoprotective agents against gastric ulcer [55]. As stated by Zhao, et al. [56] and Liu, et al. [36], flavonoids show an important role in gastro-protective mechanism via increasing gastric juice pH. Quercetin is a predominant flavonoid identified in our UPLC-ESI–Qtof-MS chart, along with its glycosides. The compound has proved its potency as an antiulcer agent towards Ethanol-induced gastric ulcer and the mechanism was possibly elucidated via Nrf2/HO1 and HMGB1/TLR4/NF-κB pathways, along with identification of the relation between them. The Nrf2 signaling pathway has an upper hand in the cell defense system as an antioxidant, along with its downstream antioxidant enzymes HO1 and CAT [54]. The antioxidant properties of flavonoids, which were previously reported in the literature [57], may be attributed to preserving epithelial integrity, leading to conservation of the upper gastrointestinal tract mucosa against gastric acidity [54]. Former studies described the influence of flavonoids, particularly quercetin, on the Nrf2 pathway in the liver [58], in the brain to improve cognitive functions [59], and in cardiomyopathy [60]. Another study carried out by Zhou et al., 2020 [61] revealed the potential of natural products, particularly gallic acid, in elevating Nrf2 and HO1, leading to intensifying the shielding effect against ethanol-induced gastric ulcer. 

Quercetin in particular has an impact on the HMGB1-TLR4-NF-κB signaling pathway, which has been linked to myocardial ischemia-reperfusion injury [62]. Consequently, in their study on the anti-ulcerogenic activity of c-phycocyanin over downregulation of HMGB1/NF-κB signalling pathway, Alzokaky, et al. [22], reported this pathway. Previous studies have connected the Nrf2 pathway to the HMGB1 pathway in a number of diseases, demonstrating that Nrf2 is involved in controlling the proinflammatory cytokine HMGB1’s activity [63,64]. To demonstrate that the ethanol effect on HMGB1 was inversely connected with that of Nrf2 for ethanol-induced gastric ulcer, Badr, et al. [65] carried out a study to support our findings.

## 5. Conclusions

This ongoing study is considered the first examination of the prospect of BIEE against ethanol-induced gastric ulcer. BIEE significantly reduced gastric damage, preserved GSH content, diminished gastric MDA level and stimulated gastric healing, to a lesser extent, than standard drug, omeprazole. Moreover, antiulcer activity was confirmed by diminishing histopathological changes and inflexing leucocytes. Indicating its antioxidant effect, pre-treatment with *B. indica* EtOH extract inhibited the increase of TLR-4, Nrf2 level and reduction of IL-1β expression via suppression of NF-κB. The antiulcer activity of the plant was attributed to its content of flavonoids, lipids and triterpenoidal saponins, identified through UPLC-ESI–Qtof-MS. Consequently, our findings stand conclude that BIEE is a potential agent and a new line for the treatment of gastric ulcer. The dose–response relationship and the proper dosage for achieving therapeutic benefits need to be better understood through additional research. Standardization and testing of individual compounds are necessary steps in further study to demonstrate the effects of active metabolites in extract.

## Figures and Tables

**Table 1:** Total pathological score recorded in the normal and *B. indica* EtOH extract treated groups.

Groups	Overall Pathologic Score
Normal control group	0.30 ^e^ ± 0.15
Ethanol-induced group	6.70 ^a^ ± 0.15
Omeprazole group	1.30 ^d^ ± 0.36
*B. indica* EtOH extract (25 mg/kg b.w)	2.50 ^c^ ± 0.42
*B. indica* EtOH extract (50 mg/kg b.w)	3.70 ^b^ ± 0.36
*B. indica* EtOH extract (100 mg/kg b.w)	1.30 ^d^ ± 0.36

Data presented as mean ± SE. ^a, b, c, d^ and ^e^ Means with different superscripts within the same column differ significantly at *p* < 0.0001.

**Table 2:** Results of TLR4 and Caspase-3 expressions in the gastric tissues of the normal and treated groups.

Groups	TLR4 Expression (% of Positive Cells/HPF)	Caspase-3 Expression (% of Positive Cells/HPF)
Normal control group	0.80 ^d^ ± 0.20	0.66 ^d^ ± 0.21
Ethanol-induced group	2.60 ^a^ ± 0.22	2.80 ^a^ ± 0.13
Omeprazole group	0.90 ^c,d^ ± 0.23	1.20 ^c,d^ ± 0.20
*B. indica* EtOH extract (25 mg/kg b.w)	1.20 ^b^ ± 0.13	1.60 ^b^ ± 0.22
*B. indica* EtOH extract (50 mg/kg b.w)	1.20 ^b^ ± 0.29	1.50 ^b,c^ ± 0.22
*B. indica* EtOH extract (100 mg/kg b.w)	0.50 ^d^ ± 0.16	0.90 ^c,d^ ± 0.23

Data presented as mean ± SE. ^a, b, c, d^ Means with different superscripts within the same column differ significantly at *p* < 0.0001.

**Table 3:** Metabolites identified in *Bassia indica* EtOH extract as analyzed via UHPLC-ESI-QToF/MS in positive ionization mode.

No	RT (min)	Mol. Ion	Molecular Formula [M + H]^+^	Error (ppm)	Name	Class	MS/MS
1	1.02	118.0864	C_5_H_12_NO_2_^+^	−1.3	Valine	Amino acid	--
2	1.04	205.082	C_7_H_13_N_2_O_5_^+^	−0.3	Glutamylglycine	Amino acid	187, 169
3	2.70	328.1379	C_15_H_22_NO_7_^+^	3.7	Fructosyl phenylalanine	Amino acid	310, 166
4	4.00	1081.314	C_48_H_57_O_28_^+^	−9.7	Cyanidin-*O*-hexosyl coumaryl-trihexoside	Flavonoid	757, 595, 449, 325, 287
5	4.10	773.2123	C_33_H_41_O_21_^+^	1.6	Quercetin-*O*-hexosyl-hamnosyl-hexoside	Flavonoid	611, 465, 303
6	4.20	787.2292	C_34_H_43_O_21_^+^	0	Isorhamnetin-*O*-rhamnosyl-di-hexoside	Flavonoid	641, 479, 325, 317, 303
7	4.30	935.2677	C_39_H_51_O_26_^+^	−1.5	Quercetin-*O*-rhamnosyl-tri-hexoside	Flavonoid	611, 465, 303
8	4.31	757.2194	C_33_H_41_O_20_^+^	−1.1	Kaempferol-*O*-rhamnosyl -di-hexoside	Flavonoid	611, 449, 287
9	4.60	949.2825	C_40_H_53_O_26_^+^	−0.6	Isorhamnetin-*O*-rhamnosyl-tri hexoside	Flavonoid	625, 479, 317
10	4.80	597.1458	C_26_H_29_O_16_^+^	−1.3	Quercetin-*O*-pentosyl-hexoside	Flavonoid	465,303
11	5.20	611.1606	C_27_H_31_O_16_^+^	0.1	Quercetin-*O*-rhamnosyl-hexoside	Flavonoid	465,303
12	5.60	465.103	C_21_H_21_O_12_^+^	−0.4	Quercetin-*O*-hexoside	Flavonoid	303
13	6.10	595.1657	C_27_H_31_O_15_^+^	0	Kaempferol-*O*-hexosyl-rhamnoside	Flavonoid	449, 287
14	6.40	625.1757	C_28_H_33_O_16_^+^	0.9	Isorhamnetin-*O*-rhamnosyl -hexoside	Flavonoid	479, 317
15	6.80	581.1864	C_27_H_33_O_14_^+^	0.2	Naringenin-*O*-hexoside	Flavonoid	435, 273
16	7.00	449.1076	C_21_H_21_O_11_^+^	0.6	Kaempferol-*O*-hexoside	Flavonoid	287
17	7.40	479.1179	C_22_H_23_O_12_^+^	1	Rhamnetin-*O*-hexoside	Flavonoid	317
18	10.30	303.0493	C_15_H_11_O_7_^+^	2	Quercetin	Flavonoid	--
19	11.40	311.2216	C_18_H_31_O_4_^+^	0.4	Hydroxy-oxo-octadecadienoic acid	Oxylipid	293, 275
20	11.42	347.2426	C_18_H_35_O_6_^+^	0.7	Dihydroxyoctadecanedioic acid	Oxylipid	311, 293, 275
21	11.50	246.2423	C_14_H_32_NO_2_^+^	1.9	Tetradecasphinganine	Sphingolipid	--
22	11.70	334.2947	C_18_H_40_NO_4_^+^	1.4	Dihydroxy-sphinganine	Sphingolipid	--
23	11.80	295.2261	C_18_H_31_O_3_^+^	2.1	Hydroxy octadecatrienoic acid	Oxylipid	277, 259, 241
24	12.30	274.2735	C_16_H_36_NO_2_^+^	2.2	Hexadecasphinganine	Sphingolipid	--
25	12.60	318.2996	C_18_H_40_NO_3_^+^	2	Phytosphingosine	Sphingolipid	256
26	12.60	487.3414	C_30_H_47_O_5_^+^	0.8	Bassic acid	Triterpenes	469, 441, 395
27	13.10	302.3046	C_18_H_40_NO_2_^+^	2.6	Sphinganine	Sphingolipid	--
28	13.30	331.2833	C_19_H_39_O_4_^+^	2.9	Glyceryl palmitate	Oxylipid	313, 239
29	13.90	330.336	C_20_H_44_NO_2_^+^	1.9	Eicosasphinganine	Sphingolipid	--
30	14.10	356.352	C_22_H_46_NO_2_^+^	0.9	*N*-Oleyldiethanolamine	Nitrogenous lipid	--
31	15.30	295.2256	C_18_H_31_O_3_^+^	3.8	Hydroxy octadecatrienoic acid isomer	Oxylipid	277
32	15.60	284.2948	C_18_H_38_NO^+^	0.1	Octadecanamide	Nitrogenous lipid	261, 252
33	15.70	279.2315	C_18_H_31_O_2_^+^	1.4	Octadecatrienoic acid	Oxylipid	261
34	15.90	299.2573	C_18_H_35_O_3_^+^	2.5	Hydroxyoctadecenoic acid	Oxylipid	281, 263
35	16.00	272.2609	C_16_H_34_NO_2_^+^	−9.3	Aminohexadecanoic acid	Nitrogenous lipid	--
36	16.40	254.248	C_16_H_32_NO^+^	−0.7	Hexadecenamide	Nitrogenous lipid	237, 219
37	16.70	279.2311	C_18_H_31_O_2_^+^	2.7	Octadecatrienoic acid isomer	Oxylipid	263, 245
38	16.90	310.3097	C_20_H_40_NO^+^	2.5	Eicosenamide	Nitrogenous lipid	291
39	16.93	300.2891	C_18_H_38_NO_2_^+^	2.1	Sphingosine	Sphingolipid	283
40	17.30	256.2628	C_16_H_34_NO^+^	2.7	Palmitamide	Nitrogenous lipid	--

## Data Availability

Data are available upon reasonable request.

## Supplementary Materials

The following supporting information can be downloaded at: https://www.mdpi.com/article/10.3390/antiox12061263/s1.
