# Supplementary material for: Metabolites Profiling and Bioassays Reveal Bassia indica Ethanol Extract Protective Effect against Stomach Ulcers Development via HMGB1/TLR-4/NF-κB Pathway
Source: Antioxidants (Basel). 2023 Jun 12;12(6):1263. doi: 10.3390/antiox12061263 (PMC10294802; doi:10.3390/antiox12061263)
Supplement: Supplementary file 1 [file antioxidants-12-01263-s001.zip › antioxidants-2423101-supplementary.pdf]

antioxidants

Supporting data

Article

Metabolites Profiling and Bioassays Reveal *Bassia indica* Ethanol Extract Protective Effect against Stomach Ulcers Development via HMGB1/TLR-4/NF-κB Pathway

Zeinab A. El-Gendy[1], Rehab F. Taher[2], Abdelbaset M. Elgamal[3], Ahmed Serag[4], Azza Hassan[5], Gehad A. Abdel Jaleel[1], Mohamed A. Farag[6], Abdelsamed I. Elshamy[2,*]

[1] Department of Pharmacology, medical research and clinical studies Institute, National Research Centre, Dokki, Giza, Egypt; dr.zeinabadel@gmail.com (Z.A.E-G.) gehad_abougharam@yahoo.com (G.A.A.)
[2] Department of Natural Compounds Chemistry, National Research Center, 33 El Bohouth St., Dokki, Giza 12622, Egypt; rehabfikrytaher@gmail.com
[3] Department of Chemistry of Microbial and Natural Products, National Research Centre, 33 El-Bohouth St., Dokki, Giza 12622, Egypt
[4] Pharmaceutical Analytical Chemistry Department, Faculty of Pharmacy, Al-Azhar University, Cairo 11751, Egypt; ahmedserag777@hotmail.com
[5] Department of Pathology, Faculty of Veterinary Medicine, Cairo University, Egypt; azzahassa1999@gmail.com
[6] Pharmacognosy Department, Faculty of Pharmacy, Cairo University, Kasr el Aini St., Cairo 11562, Egypt; mohamed.farag@pharma.cu.edu.eg
* Correspondence: algamalgene@yahoo.com (A.M.E.); elshamynrc@yahoo.com (A.I.E.); Tel.: +20-100-155-8689 (A.M.E.); +20-100-552-5108 (A.I.E)

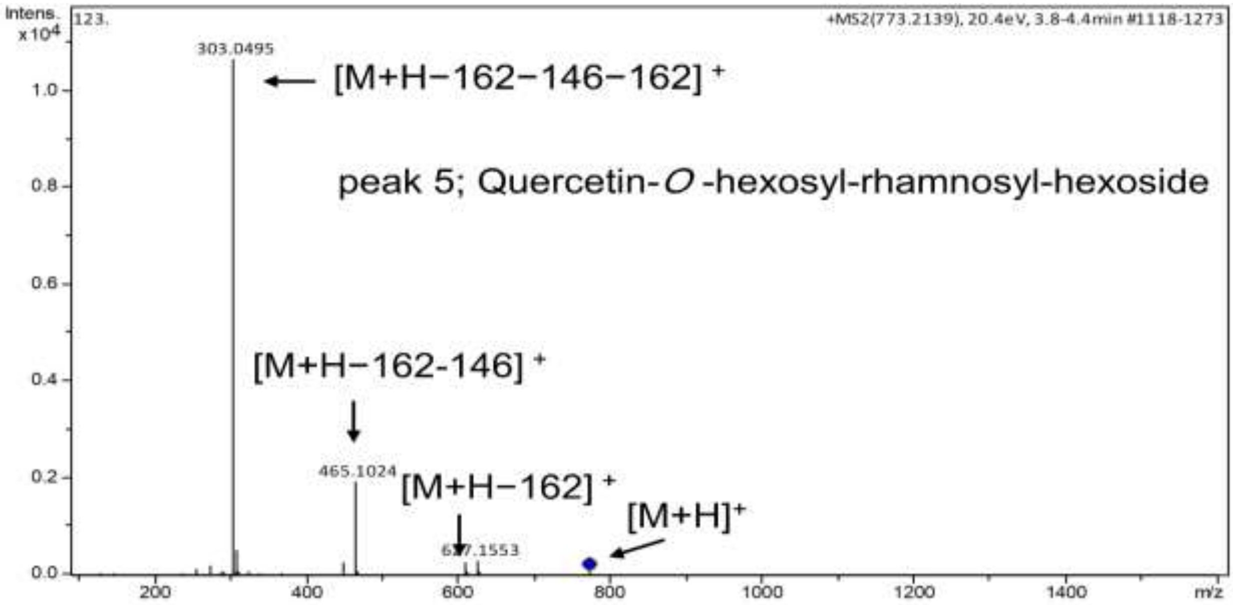

Figure S1: ESI-MS/MS spectrum of quercetin-*O*-hexosyl-rhamnosyl-hexoside (peak 5; $C_{33}H_{41}O_{21}{}^{+}$) in the positive ion mode.

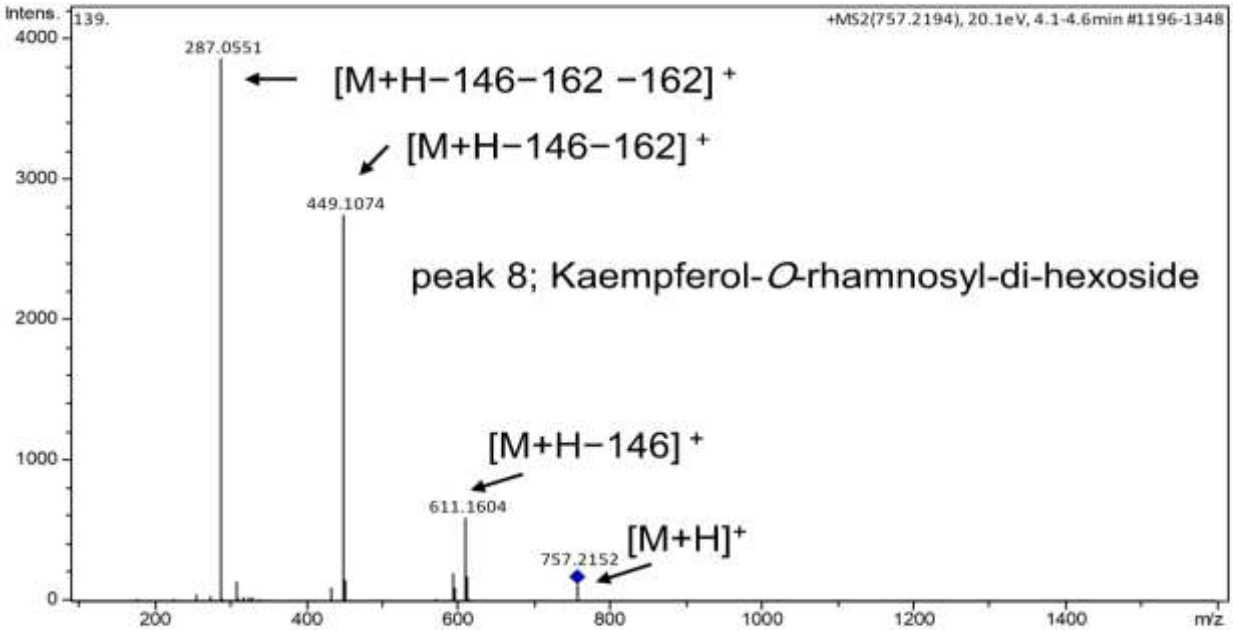

Figure S2: ESI-MS/MS spectrum of kaempferol-*O*-rhamnosyl-di-hexoside (peak 8; $C_{33}H_{41}O_{20}{}^{+}$) in the positive ion mode.

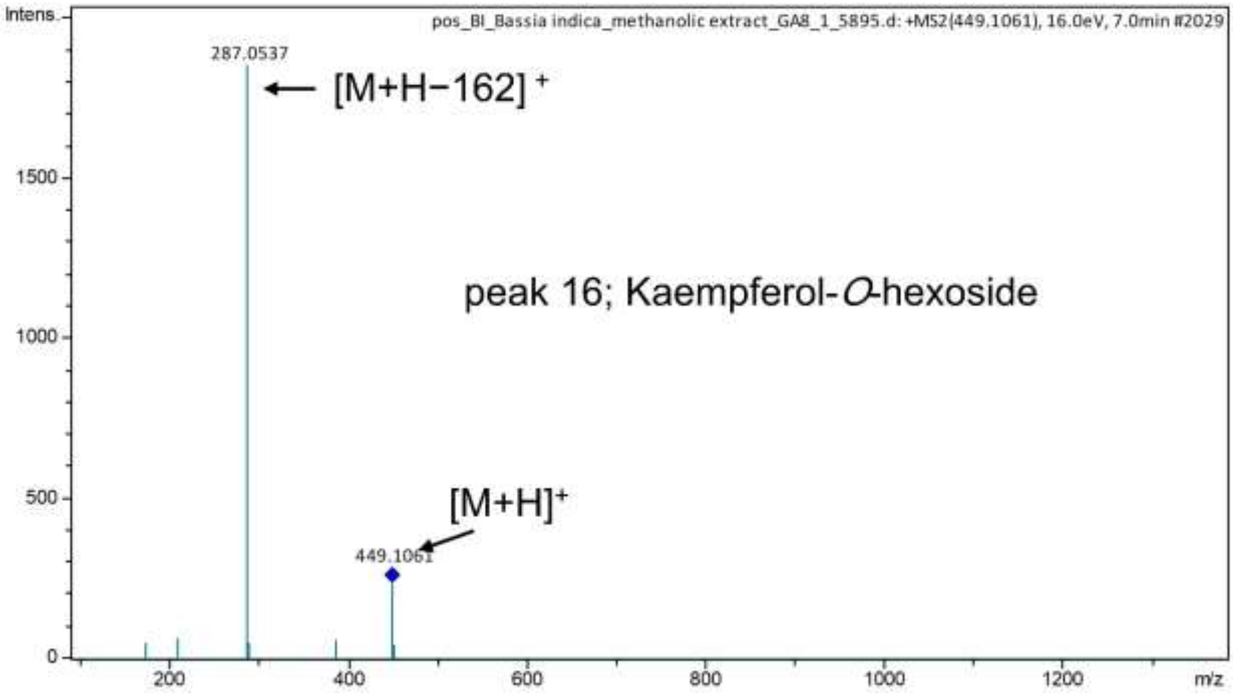

Figure S3: ESI-MS/MS spectrum of kaempferol-*O*-hexoside (peak 16; $C_{21}H_{21}O_{11}^+$) in the positive ion mode.

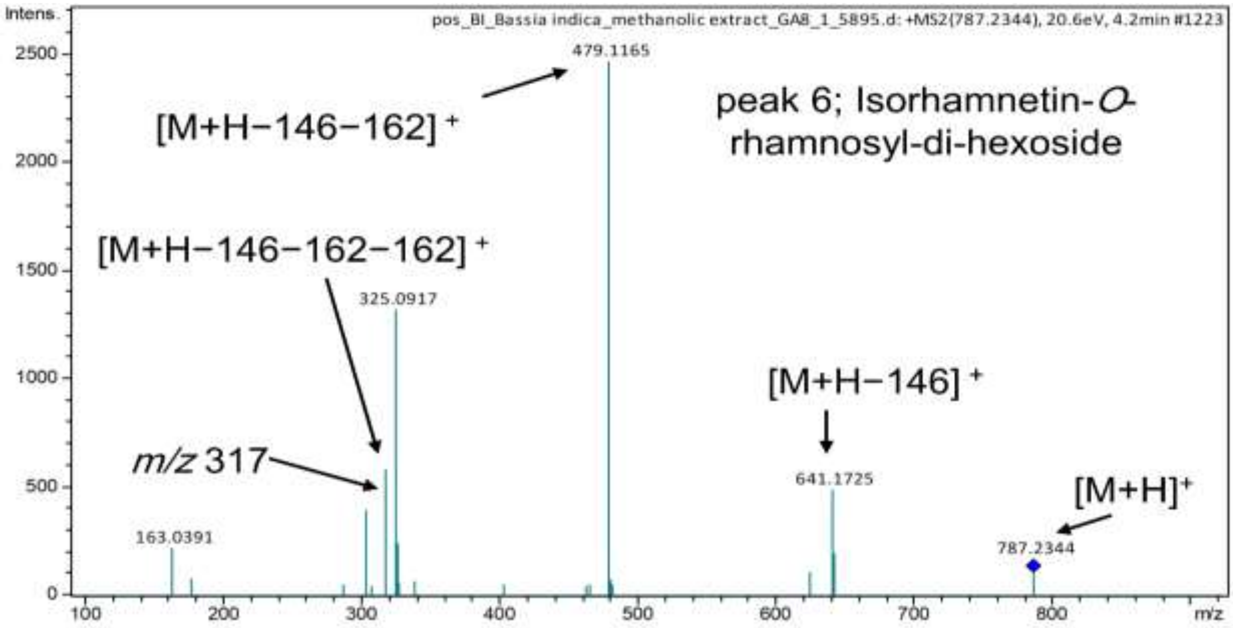

Figure S4: ESI-MS/MS spectrum of isorhamnetin-*O*-rhamnosyl-di-hexoside (peak 6; $C_{34}H_{43}O_{21}^+$) in the positive ion mode.

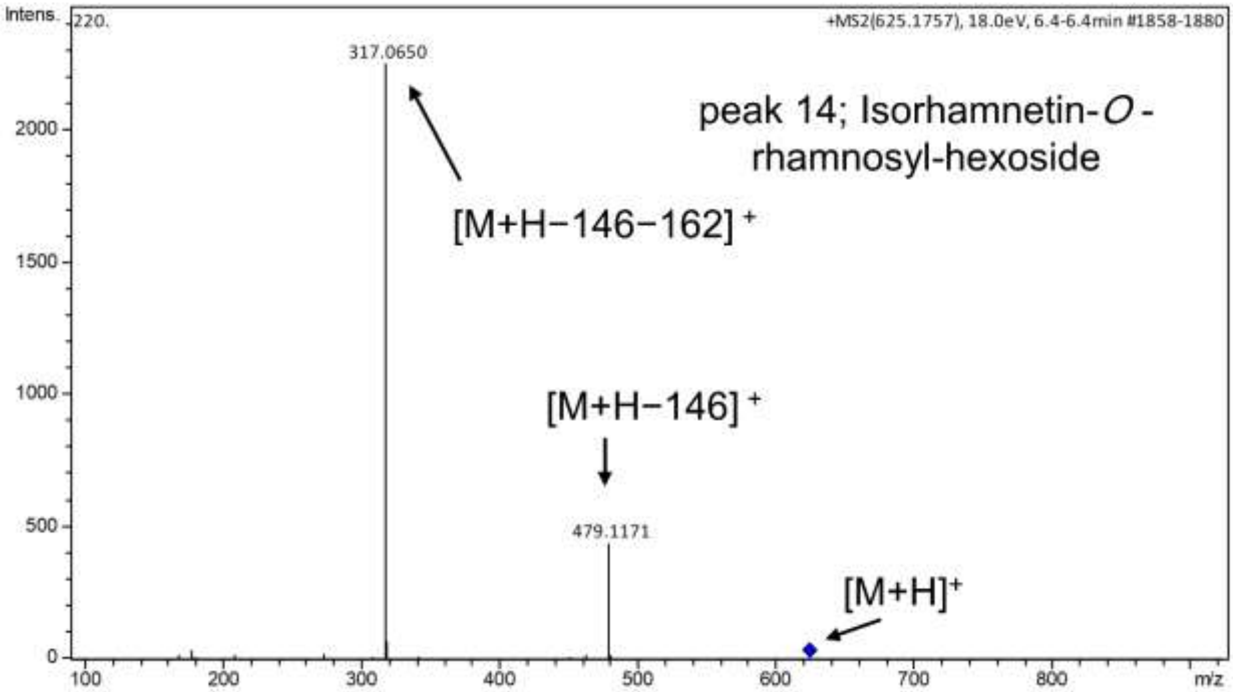

Figure S5: ESI-MS/MS spectrum of isorhamnetin-*O*-rhamnosyl-hexoside (peak 14; $C_{28}H_{33}O_{16}{}^{+}$) in the positive ion mode.
